# The role of fire disturbance on habitat structure and bird communities in South Brazilian Highland Grasslands

**DOI:** 10.1038/s41598-020-76758-z

**Published:** 2020-11-12

**Authors:** Mariana Beal-Neves, Eduardo Chiarani, Pedro Maria Abreu Ferreira, Carla Suertegaray Fontana

**Affiliations:** 1 Laboratório de Ecologia de Interações, Programa de Pós-Graduação em Ecologia e Evolução da Biodiversidade, Pontifícia Universidade Católica do Rio Grande do Sul, building 12, block C, room 111, Ipiranga Avenue 6681, Porto Alegre, RS 90619-900 Brazil; 2 Laboratório de Ornitologia, Museu de Ciências e Tecnologia, Programa de Pós-Graduação em Ecologia e Evolução da Biodiversidade, Pontifícia Universidade Católica do Rio Grande do Sul, building 40, room 112, Ipiranga Avenue 6681, Porto Alegre, RS 90619-900 Brazil

**Keywords:** Community ecology, Plant ecology, Zoology

## Abstract

Grassland ecosystems are evolutionarily linked to disturbances such as grazing and fire. These disturbances define grassland plant communities and habitat heterogeneity, which influence animal communities. We evaluated the influence of fire disturbance on plant and bird communities and on habitat structure by sampling grassland fragments with different time elapsed since the last fire event. Habitat structure was sampled using plant life forms and abiotic variables and birds were sampled through point counts. We recorded 862 bird individuals from 70 species. Intermediately-burnt sites harbor higher habitat heterogeneity and plant species richness in comparison with recently or long-burnt sites. Bird abundance and taxonomic diversity decreased linearly as time since fire increased. Finally, time since fire influenced the relative distribution of plant life forms and bird food guilds. Our results indicate that fire management should be included in the framework for conservation and sustainable use of grasslands, because it promotes habitat heterogeneity and diversity. To maintain habitat heterogeneity and the related habitat-specific bird species and functional groups, conservation efforts should maintain grassland patches under different management intensities and frequencies on a landscape level. However, studies focused on determining the periodicity with which fire management should be used are still lacking.

## Introduction

Grassland ecosystems cover approximately 40% of the world's surface^1^, harboring a unique biodiversity^2–4^. Forty of the world’s 234 Centers of Plant Diversity and 23 of the 217 Endemic Bird Areas identified by Birdlife International are inserted in grassland ecosystems^1,5^. Disturbance regimes with fire and grazing, coupled with varying climatic and geological conditions, promote high environmental diversity, different niche opportunities, and unique evolutionary scenarios, which result in high levels of diversity and endemism^2,5–7^. Bird diversity patterns and community composition are strongly influenced by such environmental drivers^6^. Among these drivers, vegetation structure and the directly related habitat complexity are key determinants of grassland bird community structure^8–10^. The frequency and/or intensity of disturbances such as fire are key determinants of grassland habitat structure^11,12^. However, there is a gap of information about how fire disturbance can influence bird community patterns.

Grassland ecosystems, as well as the biodiversity they harbor, have a close relationship with disturbance regimes^1,5,13–17^. Disturbances are events that can disrupt any ecological level of organization, environmental component or biological cycle of organisms^18^. Considering grasslands, disturbance can be more simply characterized as any event that leads to the removal of biomass from the system^19^. This biomass removal is carried out by two intrinsic and pervasive disturbances in grasslands worldwide: native, naturalized or introduced herbivores^1,13,20,21^, and fire^22,23^. Specifically, fire removes senescent plant tissue, recycles soil nutrients^1^, and increases plant species richness in comparison with unmanaged grasslands^14,24^. The frequency of fires in grasslands varies with climate, but also with choice of management^1,25–27^.

Although the effect of fire in grassland-associated fauna tends to be species-specific, the majority of the responses of grassland animal diversity to fire is linked to habitat heterogeneity (that is, the structure of grassland plant communities), resource availability, and interspecific interactions (e.g.,^9,28–32^). Plant and animal species that inhabit fire-prone environments have adaptations to this type of disturbance^33^. For example, some plant species require fire for the breakdown of seed dormancy^34,35^, while others have underground structures adapted for regrowth after burning^36,37^. Therefore, rather than being a threat to grassland ecosystems, fire is a major driver of grassland dynamics and physiognomy, promoting and maintaining diversity^27^. In plant communities, the mechanism behind this process is related to differential species selection (i.e., species with traits related to fire resistance/resilience) and to the ‘break of dominance’ , i.e., the reduction of aboveground biomass of large tussock-forming species that dominate grasslands in the absence of disturbance^14,26,38^. Ultimately, fire promotes grassland habitat heterogeneity through these processes. In the absence of disturbances, grassland structure becomes homogeneous^14,39–41^, mostly dominated by tall, erect grasses^28,41^, and shrubs^41–44^. This structural simplification results in biodiversity losses for plant and animal species^14,41^. Several studies have used a functional approach to measure patterns of community reorganization after disturbances^45,46^. For plant communities, a reduced set of functional traits such as life forms can be used to detect changes in community structure that result from different disturbance regimes^47^. Birds are sensitive to such changes in plant communities. For example, bird communities are affected by forest structure considering taxonomic and functional descriptors^48^. Similar responses of the bird communities could be expected regarding shifts in grassland habitat structure that follow shifts in their disturbance regime^9,10^.

The role of disturbances as drivers of biodiversity patterns has been one of the central issues in ecology for a long time. Diversity-disturbance relationships (DDR) can be extremely variable considering both strength and shape^49^. In general terms, any significant response of diversity to disturbance intensity and/or frequency can be monotonic (either positive or negative), U-shaped, or peaked (hump-shaped). Peaked relationships are commonly linked to the intermediate disturbance hypothesis (IDH;^50–52^). Even though the IDH has been recently challenged in what regards its underlying premises and mechanisms^53^, peaked relationships do seem to be a recurrent pattern in DDR, especially when disturbances are intrinsic to the study system^49^. Peaked responses to disturbance by grazing are arguably the most common DDR in highly productive grassland ecosystems with long coevolutionary history with grazing animals^54,55^. However, the mechanisms and observed effects of fire on grassland diversity can be different from those produced by grazing (e.g.,^56–58^). Although the effect of fire disturbance on plant diversity has been addressed rather extensively in the literature (e.g.,^59–62^), fire-diversity relationships are often tested with monotonic models, neglecting the possible peaked response. Moreover, the strength and shape of this DDR have been largely overlooked for bird communities (but see^30,63^).

Overall diversity and relative abundance of avian species decline as fire-suppressed grasslands become denser (i.e., dominated by a few tussock species) which, coupled with litter accumulation, makes foraging difficult for ground-feeding birds^64^. Recently-burnt grassland patches from South Africa, with short and litter-free vegetation, showed significantly higher values of bird species richness and diversity^9^. The abundance of grassland arthropods, which are an important food source for many bird species, increases after burning. This seems to be one of the indirect mechanisms behind the influence of fire over bird communities^9,65^. Raptors (Accipitridae and Falconidae) are attracted to newly-burned areas by the availability of prey^66^. In the Brazilian Cerrado, birds made direct use of the resources provided by firebreak areas, indicating that prescribed fires promote feeding opportunities for many bird species, among other animal taxa^67^. When fire regimes comprise either very frequent or very rare fire events, the availability of grass seeds for granivores may also be affected via the reduction of plant and seed density^68^.

There is also evidence that patterns of bird communities from South Brazilian Highland Grassland are dependent on fire disturbance, although no comprehensive community-wide study has been carried out in the system. Certain species (including endemic, migratory, and/or extinction-threatened ones) tend to be more frequent and abundant in frequently-burnt areas, where vegetation structure is overall shorter (e.g., *Cinclodes pabsti*, *Anthus nattereri* and *Xanthopsar flavus*), whereas a different set of species seem to select areas with higher vegetation, where fire frequency is lower (e.g., *Scytalopus iraiensis*, *Limnoctites rectirostris Sporophila melanogaster*, and *S. beltoni*)^69,70^. In fact, frequent fires seem to negatively affect species that are dependent on ‘tallgrass structures’, such as the Lesser Grass-Finch—*Emberizoides ypiranganus* and the Black-bellied Seedeater—*Sporophila melanogaster*, due to loss of suitable habitat to nest, forage, and seek refuge^71^. Finally, fire may also have direct negative effects on the reproductive success of grassland birds if it takes place during the breeding period, leading to nest losses^72^ or increasing rates of nest predation and brood parasitism^73,74^. Notwithstanding, most of the continuous well-preserved grassland areas in Southern South America are traditionally managed with fire disturbance as part of the cattle raising system^2,15,17,75^. Therefore, basic knowledge on how multiple taxonomic groups respond to management is fundamental. Such studies are especially important for the Brazilian reality, since management with fire and grazing is still a controversial issue, especially in protected areas^76,77^.

Here we evaluated the effect of fire frequency, described by the time elapsed since the last fire event (hereafter ‘time since fire’, as seen in^14,17,26^), on habitat structure (i.e., plant communities) and bird community descriptors in Highland Grasslands from South Brazil. Based on the premises that (i) fire disturbance promotes habitat heterogeneity in grasslands by breaking the species dominance, and (ii) habitat heterogeneity is a key driver for bird community patterns, we expect the following results. In comparison with recently-burnt sites, sites with longer time since fire will present: (1) higher vegetation height, (2) lower habitat heterogeneity, (3) lower plant species richness, (4) lower bird taxonomic and functional diversity, and (5) different bird food guild composition. In addition, we tested the direct influence of habitat structure on bird taxonomic and functional community patterns.

## Materials and methods

### Sampling sites

**Figure 1:**
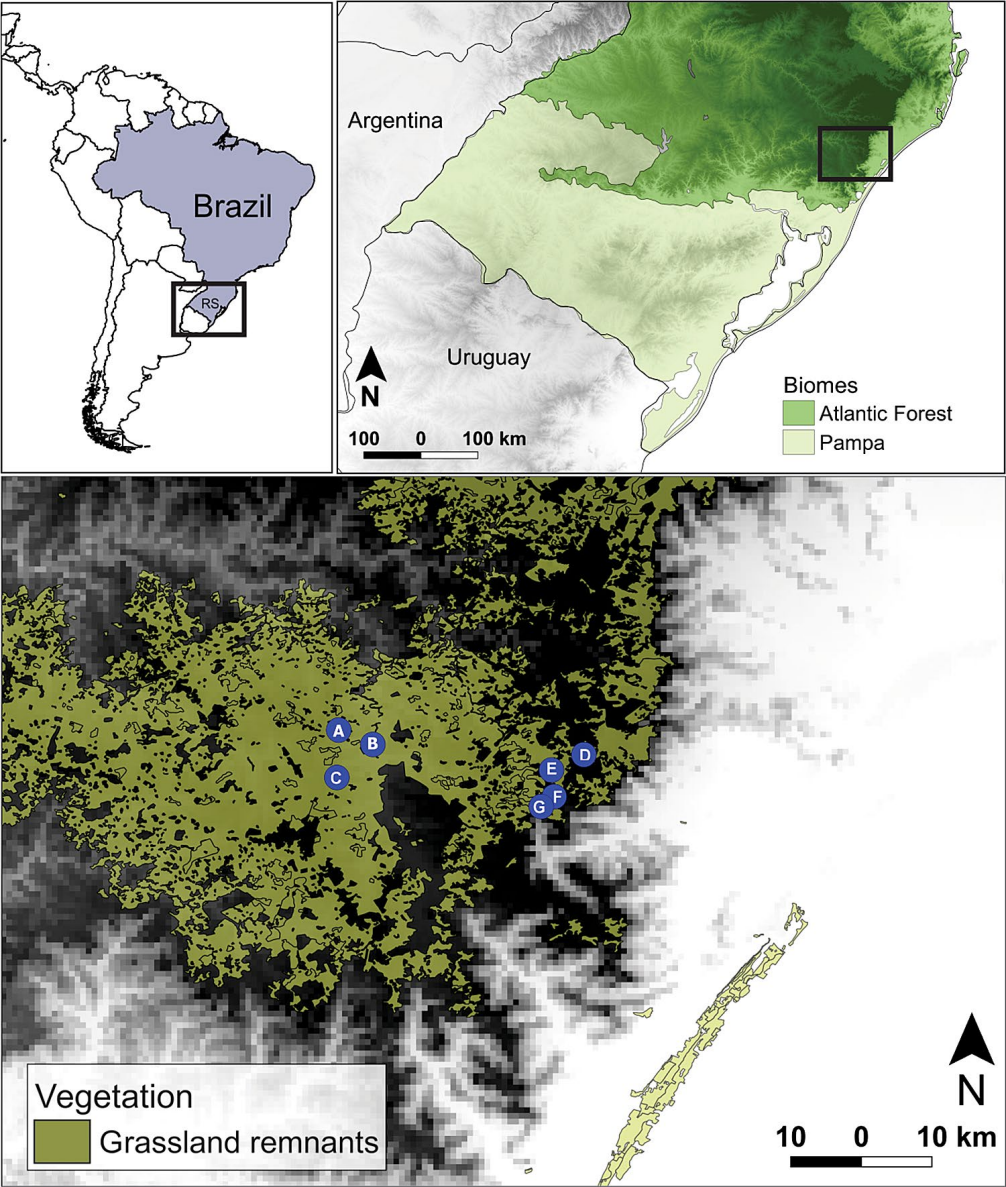
Sampling sites. Location of the seven sampling sites in South Brazilian Highland Grasslands (blue circles **A**–**G**). Sites (**A**) and (**C**) are in the immediate vicinity of Tainhas State Park, in private farms, while site (**B**) is inside of this protected area. Site (**D**) is inside of Serra Geral National Park and (**E**–**G**) are inside Aparados da Serra National Park. Respective time since fire (months) of each site: (**A**) (5), (**B**) (17), (**C**) (5), (**D**) (180), (**E**) (16), (**F**) (180), (**G**) (3). Altitude variation is represented by the gradient between white (sea level) and black (maximum altitude: 1,100 m above sea level). We used the software QGIS^118^, version 3.4.12 to generate this figure (https://qgis.osgeo.org).

The study sites were located in South Brazilian Highland Grasslands (in the state of Rio Grande do Sul, Brazil 29º05′S, 50º05′W), inserted in the Atlantic Forest biome, where there is a physiognomy of grassland-forest mosaics (Fig. 1; see^75^ and^78^ for a review on plant and bird communities from these ecological systems, respectively). We selected seven sites (A–G; Fig. 1) with natural grassland vegetation that comprised a chronosequence of different fire disturbance histories. This chronosequence describes the time elapsed since the last fire event in each site (‘time since fire’), i.e., the amount of time that plant and bird communities in each site had to reassemble after the reset promoted by fire. This terminology was already applied in previous studies of fire disturbances in South American grasslands^14,17,26^. The time since fire varied from 3 to 180 months (see Fig. 1), and was either monitored by the researchers or reported by landowners and park managers. Sites A and C are in the immediate vicinity of Tainhas State Park, in private farms, while site B is inside of this protected area. Site D is inside of Serra Geral National Park and sites E–G are inside Aparados da Serra National Park (Fig. 1). All sites have been under cattle grazing for many decades. A recent regional classification of South Brazilian grasslands indicated ‘highland grasslands’ as a discrete unit that share not only soil features and plant community structure, but similar management as well^79^. Natural grasslands in the region are historically used as the primary forage source for free-range cattle ranching. Grazing pressure is usually very low, with stocking rates of less than 1.0 animal unit (450 kg of live weight) per hectare^75^. These low stocking rates are based on the low carrying capacity of the grasslands during winter, but are maintained throughout the year. This leads to the accumulation of large quantities of dead biomass and, ultimately, to use of fire to ‘reset’ the system with new (and more palatable) plant biomass^26,75,80^. Because part of the land of the protected areas are still under private management (i.e., federal or state authorities are still in the process of paying and reclaiming the ownership of the land from original landowners), grazing pressure is similar in farms and protected areas. In addition, even in protected areas that are officially owned and managed by federal or state authorities, cattle grazing by animals from neighboring private properties is not uncommon. We did not directly estimate grazing pressure in the sampling sites, because we assumed that the low and similar stocking rates would have similar effects on biological descriptors in all sites. In addition, we assumed that the effect of time since fire would be stronger and more detectable in comparison with the effect of eventual small variances in grazing pressure across sites.

### Bird sampling

We sampled birds during the peak of breeding season, between December 2017 and February 2018, using point counts of 10 min and 80 m radius^81^. Point counts were carried out under appropriate weather conditions and by the same observer in the mornings (sunrise until 10:00 a.m.). The number of points was proportional to the size of the grassland fragment at each site, and points were allocated at least 300 m apart. They were allocated systematically in sampling sites, the sampling effort in these different habitats was proportionate to their spatial representativeness in each site and distributed to cover as much of the grassland-covered area as possible. They were always located in open areas at least 150 m from the edges of other vegetation types (e.g., forests) or from fences and isolated trees. A total of 83 point counts were performed, distributed among sites as follows: A, B (14), C, E (12), D (11), F, G (10). Each point count was sampled twice, with a difference of two months. To avoid double counts, we used only the maximum number of individuals recorded in the two sampling events of each point. We used bird community mean values for each site (A-G) in all analyses that follow.

### Plant and habitat structure sampling

Vegetation sampling was carried out in February 2018 immediately after the sampling of birds. We classified plant species into three life forms: shrubs, forbs (i.e., non-graminoid herbs), and graminoids^41,81–84^. We used plant species richness and abundance as plant community descriptors, as well as vegetation height, and the amount (horizontal cover in percentage) of bare soil, water, and rock. Since graminoid species were the dominant component in all sampling sites, and because the species-specific estimation of abundance in graminoids can be very difficult and specialist-dependent to be accurate, we did not sample graminoid abundance. In addition, we counted all individuals of *Eryngium*, a large, thorny, bromeliad-like forb, widely used by grassland bird species for nest building and foraging^66,85^ as another descriptor of habitat structure. At each bird point count, we sampled vegetation variables in four 1 m^2^ quadrats, distributed among sites as follows: A, B (56), C, E (48), D (44), F, G (40). These quadrats were allocated from the bird point count 10 m to the north, 25 m to the west, 50 m to the south, and 75 m to the east. This approach was used to maximize the coverage of the habitat heterogeneity that may exist at each point count^85,86^. In addition, we counted all individuals of *Eryngium* and shrubs in 10 m buffers around each quadrat, so that the sampling method encompassed larger specimens and covered a larger portion of the habitat used by birds sampled in point counts.

### Data analysis

**Figure 2:**
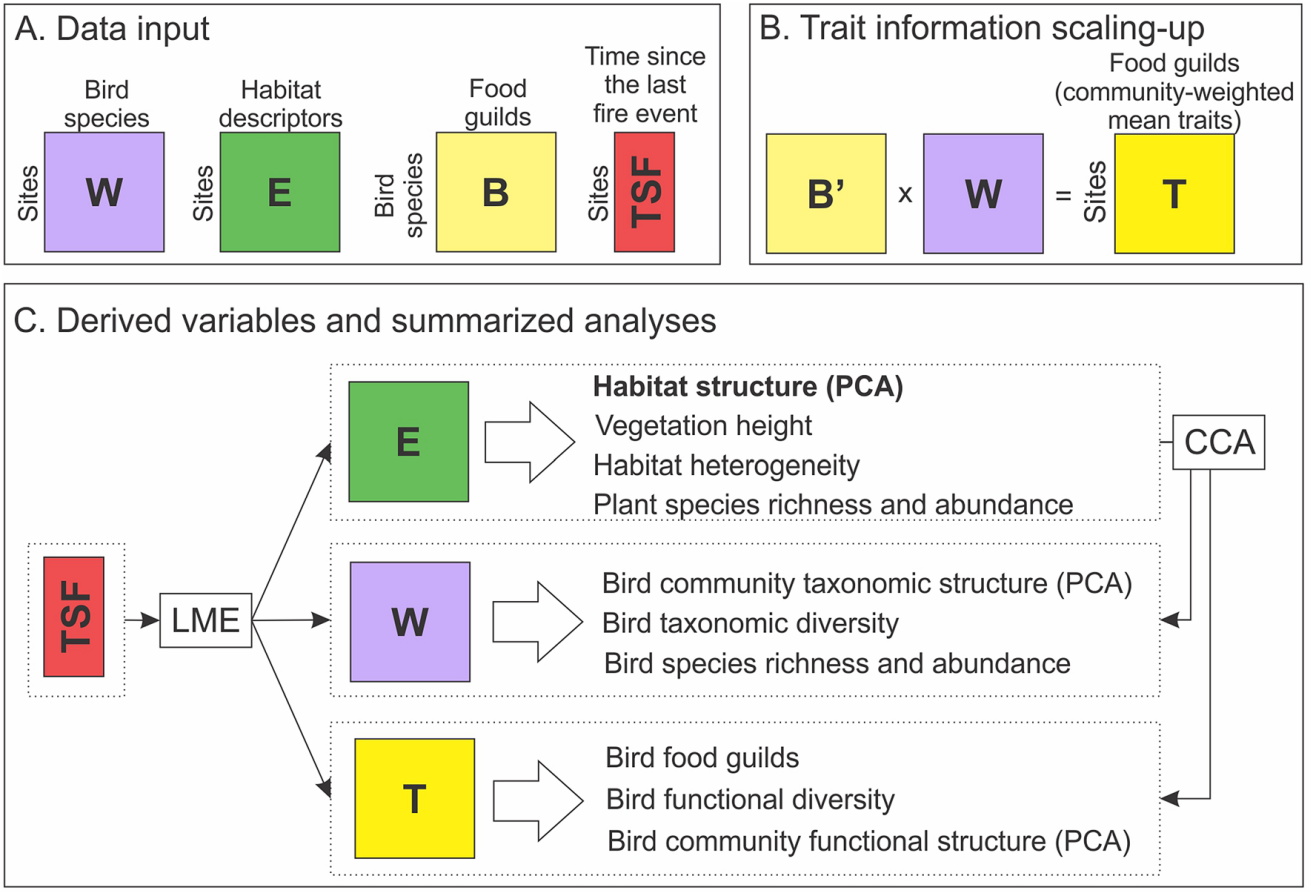
Analytical framework. (**A**) Data input. Matrix **W** of seven sites described by bird species abundance; matrix **E** of seven sites described by habitat descriptors; matrix **B** of bird species separated in food guilds; seven sites described by the time since the last fire event (TSF). (**B**) Trait information scaling-up. Matrix **T** with community-weighted mean trait values, obtained from matrix multiplication B’*W. (**C**) Derived variables and summarized analyses. TSF as independent variable for Linear Mixed-Effect Models (LME), with each dependent variable derived from data matrices. The first ordination axis of Matrix **E** (Habitat structure (PCA), in bold) was used as independent variable in separate models, with bird variables derived from matrices **W** and **T** as dependent variables. Finally, matrix **E** was used in Canonical Correspondence Analyses (CCA) with matrices **W** and **T**.

The first step in our analytical framework was to summarize plant and bird data, as well as time since fire (log-transformed for all analyses), in matrices with pooled values for each sampling site (Fig. 2A). In this paper, all analyses were carried out at the site level, and patterns related to within-site variations will be addressed elsewhere. Plant community and habitat descriptors were pooled in matrix **E** of seven sampling sites described by mean values of each variable: plant species richness (total and separate by graminoids, forbs, and shrubs); abundance (forbs, shrubs, and *Eryngium*); percentage (cover estimation in percentage) of vegetation, water, rock, and bare soil; and mean vegetation height. Abundance of shrubs and *Eryngium* was sampled in 1 m^2^ plots and in 10 m buffers. This matrix was used as a descriptor of habitat structure. Since the variables that comprised matrix **E** could show varying levels of collinearity, we used the Variance Inflation Factor (VIF) to detect multicollinearity^87^, using package ‘olsrr’^88^. We calculated VIF for all variables in matrix **E**, which resulted in values lower than 5 for all variables (which indicates insignificant inflation due to multicollinearity^89^).

Bird community data were summarized in matrix **W**, with seven sites described by mean values of species abundance (Fig. 2A). This matrix was used as a descriptor of bird community taxonomic structure. We divided bird species in six food guilds (treated as separate binary traits in all analyses): (Ne) nectarivores, (Gr) granivores, (Fr) frugivores, (In) insectivores, (Ca) carnivores, and (Om) omnivores. We consider a species as omnivore when it could be simultaneously included in three or more guilds. The sorting of species into guilds was based on the ‘Handbook of the Birds of the World Alive’^90^, and on personal field experience. Bird food guild information was summarized in matrix **B** of bird species described by six food guilds as binary functional traits (Fig. 2A). Trait information was scaled-up to the community level by matrix multiplication **B'W**, resulting in matrix **T** with community-weighted mean (CWM) trait values, which in our case represent the relative contribution of each food guild at each sampling site^91^ (Fig. 2B). This matrix was used as a descriptor of bird community functional structure.

We performed Principal Component Analyses (PCA) to extract the scores of the first ordination axis of matrices **E**, **W,** and** T** (Fig. 2C). With this, we obtained vectors describing habitat structure and bird taxonomic and functional structure, respectively. Additionally, we calculated bird taxonomic diversity using Simpson’s 1-D^92^ from matrix **W**, and bird functional diversity using Rao’s entropy based on Gower dissimilarities^93^ from matrix **T**. Finally, we estimated habitat heterogeneity by calculating Simpson’s diversity index based on the composition of plant life forms from matrix **E** (Fig. 2C). In this sense, the heterogeneity index can be interpreted similarly to the species diversity index, theoretically ranging from zero (complete dominance of one life form) to one (relative contribution of life forms evenly distributed).

All analyses were carried out in the R environment^94^, and we assumed the probabilistic threshold of 95% for statistical significance. To test most of our hypotheses, we used a statistical framework based on Linear Mixed-Effect Models^95^ (“LME”; Fig. 2C), with package ‘nlme’^96^. We built separate models with time since fire (log-transformed) as independent variable and individual variables and ordination axis from matrices **E**, **W**, and **T** as dependent variables, to test the hypotheses related to the influence of disturbance frequency on plant/habitat and bird community taxonomic and functional descriptors, respectively. Additionally, we tested the relationship between habitat structure (first ordination axis of Matrix **E**) on bird taxonomic and functional descriptors. Regression models that involve the response of CWM traits to environmental variables may render highly biased results, such as the inflation of type-I error^96–100^. To avoid this potential problem, we used the multilevel method (MLM3) proposed by ter Braak^99^ to test these specific relationships. In this method, models are corrected by weighting with Hill’s effective number (N_2_) of species’ occurrence and N2-diversity of sites, which are then combined in a sequential permutation-based test (‘max test’). The test returns two separate p-values for the model, one for sites and other for species that generated the CWM traits, and we report both in the results. Since our sampling sites were distributed in two spatially segregated areas (Fig. 1), we included the area (Tainhas and Serra Geral/Aparados area) as a random variable in all models to control for spatial autocorrelation. For each tested relationship, we built two concurrent models, considering a linear and a quadratic relationship between dependent and independent variables. We then compared each pair of concurrent models using analyses of variance with permutation and, when comparisons resulted in significant differences, we chose the best model based on the Akaike Information Criterion (AICc;^95^). When models were equivalent, we used the linear relationship. We estimated the explanatory power of all models by calculating pseudo-R^2^ (Maximum Likelihood) and p-values through comparisons with null models^95^ using package ‘rcompanion’^101^. We chose to compare between concurrent models because we wanted to evaluate if plant and bird community descriptors response to time since fire was either linear or peaked (i.e., with highest values in intermediate disturbance frequencies, as described by a quadratic model).

Bird community descriptors were separated in two dimensions for statistical analysis: taxonomic descriptors—abundance, species richness, diversity, and composition (first ordination axis of matrix **W**), and functional descriptors—functional diversity, individual CWM traits, and guild composition (first ordination axis of matrix **T**). After, to address the hypotheses related to the influence of habitat structure on bird taxonomic and functional composition, we used a Canonical Correspondence Analyses (CCA;^102,103^; Fig. 2C). The CCA was performed with matrices **W** and **T**, using matrix **E** to constrain the ordinations. To avoid overfitting, we reduced the original matrix **E** by removing all variables that showed significant collinearity. The remaining variables were mean vegetation height and abundance of forbs, shrubs, and *Eryngium* in a 10 m radius. We tested the statistical significance of the CCA models using Analysis of Variance with permutations.

## Results

We recorded 862 individuals from 70 bird species, belonging to six food guilds. Insectivores and omnivores were the most representative food guilds, corresponding to 43% and 33% of the species, respectively, followed by granivores (11%). Carnivores, nectarivores, and frugivores together comprised 13% of the bird species. Four species are globally threatened (*Anthus nattereri*, *Scytalopus iraiensis*, *Xanthopsar flavus*, and *Xolmis dominicanus*), and three are near-threatened (*Cinclodes pabsti*, *Limnoctites rectirostris*, and *Sporophila melanogaster*)^104^. Three of these species with conservation concern (*A. nattereri*, *C. pabsti*, and *X. flavus*) were restricted to recently-burned sites, whereas *S. melanogaster* was recorded in all sites but showed higher abundance in sites with longer time since fire. (See Supplementary Table S1 with matrices **W** and **T** containing complete taxonomic and functional bird data, as well as species common names and IUCN threatened species categories).

**Figure 3:**
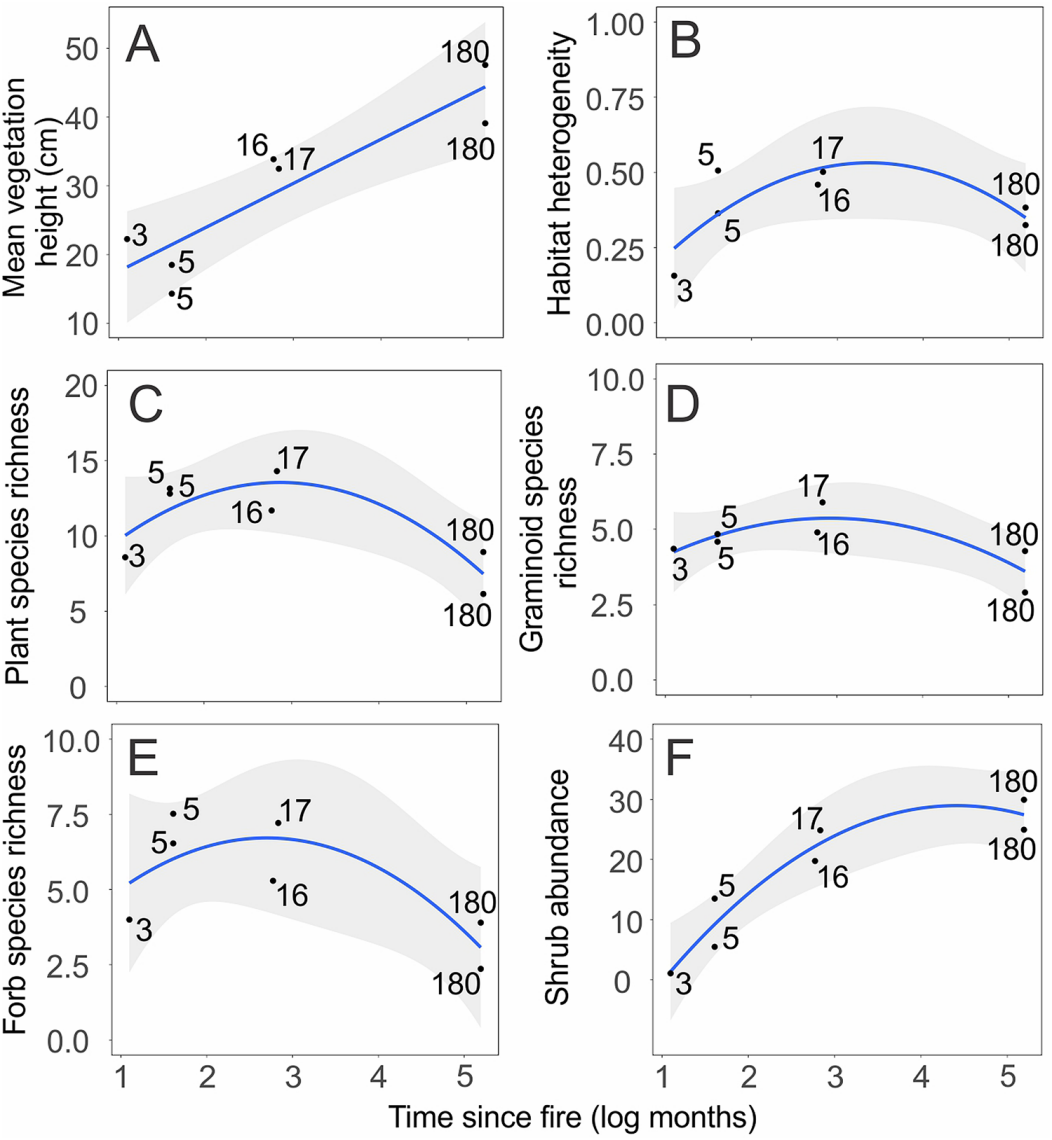
Relationship between time since fire and plant community descriptors. Time since fire as a predictor of (**A**) vegetation height. (**B**) habitat heterogeneity. (**C**) total plant species richness. (**D**) graminoid species richness. (**E**) forb species richness. (**F**) abundance of shrubs in a 10 m-radius surrounding sampling points. Time since fire log-transformed in the horizontal axis, with raw values shown in data points. Data points represent mean values derived from 332 vegetation sampling plots (four plots in each of the 83 bird point-counts).

**Figure 4:**
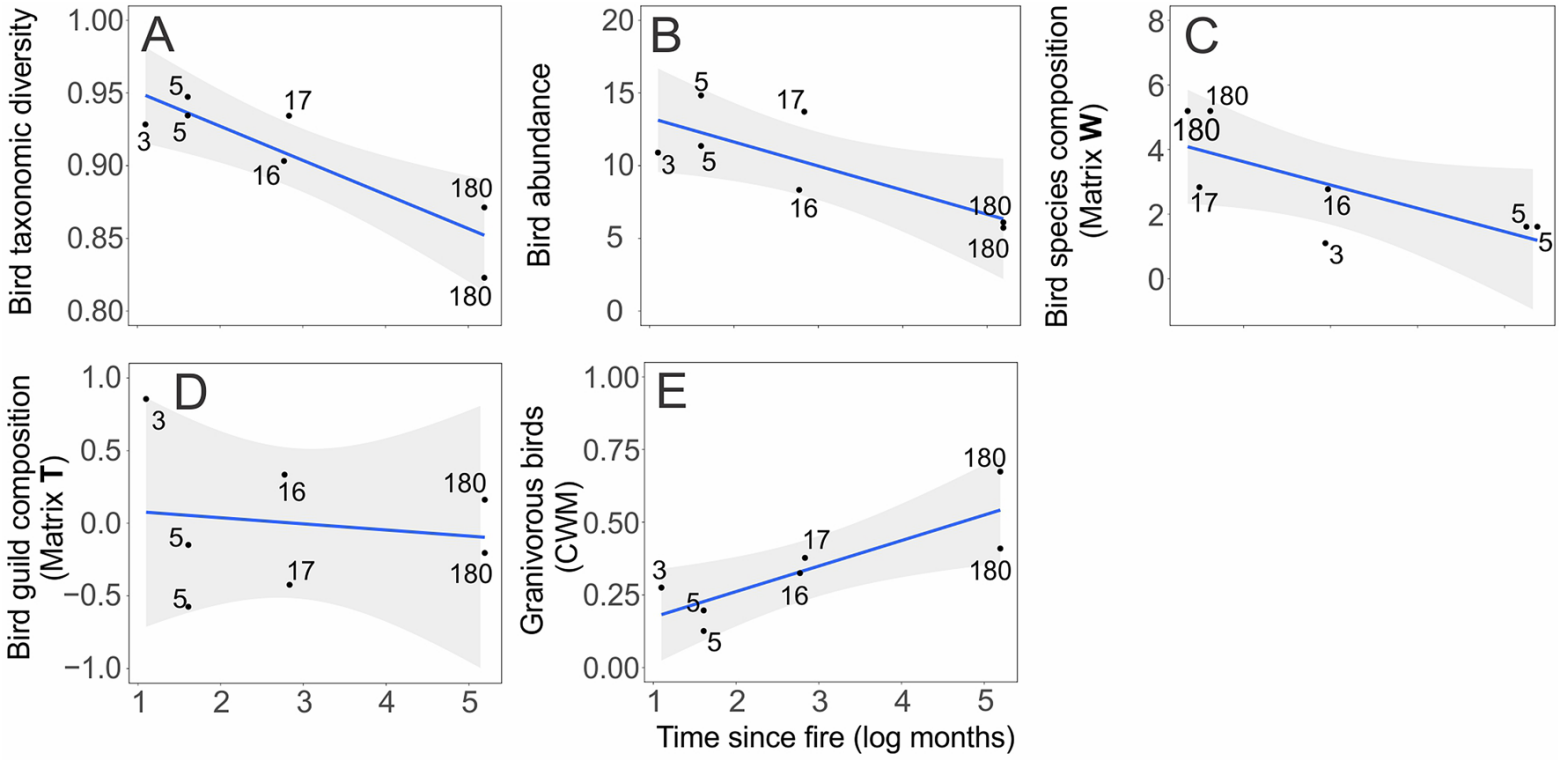
Relationship between time since fire and bird community descriptors. Time since fire as a predictor of (**A**) taxonomic diversity. (**B**) abundance. (**C**) species composition and overall community structure (first ordination axis of Matrix **W**). (**D**) functional composition (described food guilds; Matrix **T**). (**E**) proportion of granivorous birds. E represent community-weighted mean trait values extracted from Matrix **T**. Data points represent mean values derived from 83 bird point-counts. Time since fire log-transformed in the horizontal axis, with raw values shown in data points.

The relationship between plant community descriptors and time since fire fitted a quadratic relationship in all cases, with the exception of mean vegetation height, which increased linearly with time since fire (Table 1, Fig. 3). However, linear and quadratic models were equivalent regarding the response of bird community descriptors to time since fire and plant/habitat descriptors, with the exception of bird taxonomic diversity explained by habitat structure (Table 1, Fig. 4).

**Table 1:** Relationship between plant and bird community descriptors resulting from Mixed-Effects models. Simplified model syntax shown as ‘dependent variable ~ independent variable’. TSF = time since fire.

Model		Pseudo-R2	*p* value (model)	AIC	*p* value (between models)
Mean vegetation height ~ TSF	Linear	0.820	0.001	49.305	0.441
	Quadratic	0.835	0.002	50.712	
Habitat heterogeneity ~ TSF	Linear	0.030	0.593	− 2.893	0.011
	Quadratic	0.624	0.033	− 7.449	
Plant species richness ~ TSF	Linear	0.161	0.268	38.443	0.005
	Quadratic	0.733	0.010	32.416	
Graminoid richness ~ TSF	Linear	0.248	0.158	23.187	0.013
	Quadratic	0.689	0.017	19.003	
Forb richness ~ TSF	Linear	0.238	0.168	30.144	0.032
	Quadratic	0.605	0.039	27.542	
Shrub abundance (10 m-radius) ~ TSF	Linear	0.757	0.002	50.195	0.005
	Quadratic	0.921	< 0.001	44.359	
Habitat structure (Matrix **E**) ~ TSF	Linear	0.882	< 0.001	26.671	0.326
	Quadratic	0.897	< 0.001	27.707	
Bird taxonomic diversity ~ TSF	Linear	0.776	0.001	− 27.713	0.174
	Quadratic	0.828	0.002	− 27.561	
Bird abundance ~ TSF	Linear	0.593	0.012	35.840	0.543
	Quadratic	0.614	0.036	37.469	
Bird species composition (Matrix **W**) ~ TSF	Linear	0.534	0.021	19.263	0.673
	Quadratic	0.546	0.063	21.085	
Bird species composition (Matrix **W**) ~ Habitat structure (Matrix **E**)	Linear	0.741	0.002	15.144	0.123
	Quadratic	0.816	0.003	14.764	
Bird taxonomic diversity ~ Habitat structure (Matrix **E**)	Linear	0.947	< 0.001	− 28.547	0.002
	Quadratic	0.801	0.001	− 35.819	
Bird abundance ~ Habitat structure (Matrix **E**)	Linear	0.510	0.026	37.144	0.532
	Quadratic	0.536	0.068	38.753	
Bird guild composition (Matrix **T**) ~ TSF	Linear	0.490	0.030	11.247	0.721
	Quadratic	0.499	0.089	13.120

Recently-burnt sites presented lower vegetation height in comparison with sites with longer time since fire (Fig. 3A). Sites with intermediate time since fire presented the higher values of habitat heterogeneity (Fig. 2B), plant species richness (Fig. 3C), graminoid species richness (Fig. 3D), and forb species richness (Table 1, Fig. 3E). On the other hand, the abundance of shrubs was higher in sites with longer time since fire, although the relationship was better explained by a quadratic model (Table 1, Fig. 3F). Habitat structure (the first ordination axis of matrix **E**) was also explained by time since fire (Table 1). However, total plant abundance and *Eryngium* abundance were not predicted by time since fire (LME *p* = 0.088; *p* = 0.297, respectively). See Supplementary Table S2 for the complete list of habitat variables (Matrix **E**).

The relationship between bird descriptors and time since fire fitted a linear relationship in all cases. Bird taxonomic diversity and abundance were higher in recently-burnt sites (Table 1, Fig. 4A,B), but time since fire did not influence neither bird species richness (LME *p* = 0.051) nor bird functional diversity (LME *p* = 0.698). In addition, bird species composition (first ordination axis of matrix **W**) was predicted by time since fire (Table 1, Fig. 4C) and by habitat structure (first ordination axis of matrix **E**; Table 1). Habitat structure also predicted bird taxonomic diversity and abundance (Table 1). Bird community functional composition described by food guilds (CWM traits, first ordination axis of matrix **T**) was also predicted by time since fire (Table 1, Fig. 4D). The relative contribution of granivores was higher in sites with longer time since fire (MLM3 p_SITES_ = 0.012, p_SPECIES_ = 0.036, Fig. 4E).

**Figure 5:**
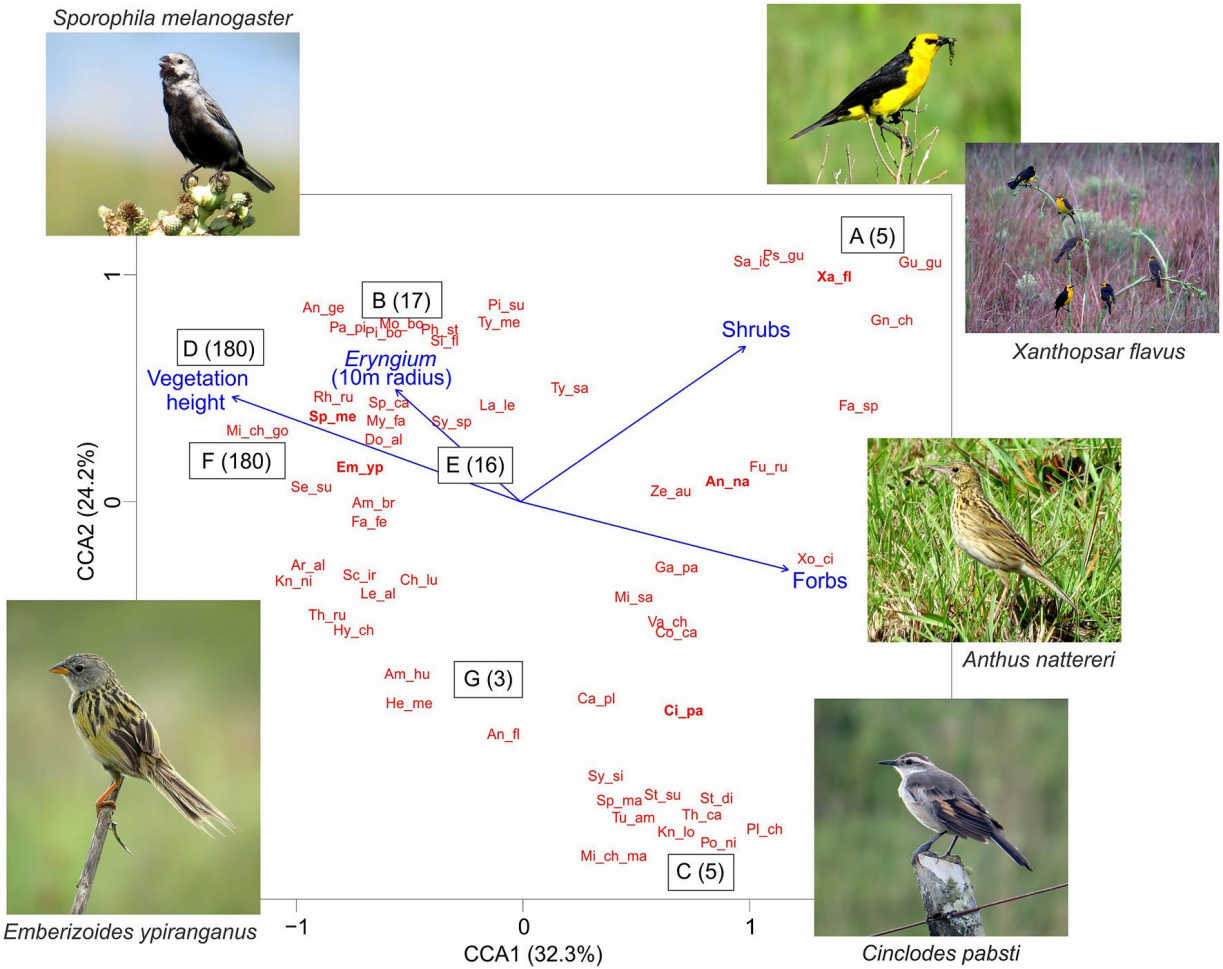
Scatterplot of a Constrained Correspondence Analyses of bird species. Constraining habitat variables shown in blue arrows: mean vegetation height and abundance of forbs, shrubs, and *Eryngium* in a 10 m radius. Sampling sites indicated by letters (as in Fig. 1) followed by time since fire. Bird species shown in red (see Supplementary Table S1 for complete species list with species common names and IUCN threatened species categories). Selected representative species (due to differential habitat use; see discussion) shown in photographs and in bold red. Respective time since fire (months) of each site: (**A**) (5), (**B**) (17), (**C**) (5), (**D**) (180), (**E**) (16), (**F**) (180), (**G**) (3). All species photos presented in the figure were taken in Tainhas State Park, Jaquirana, Rio Grande do Sul, Brazil by Eduardo Chiarani.

**Figure 6:**
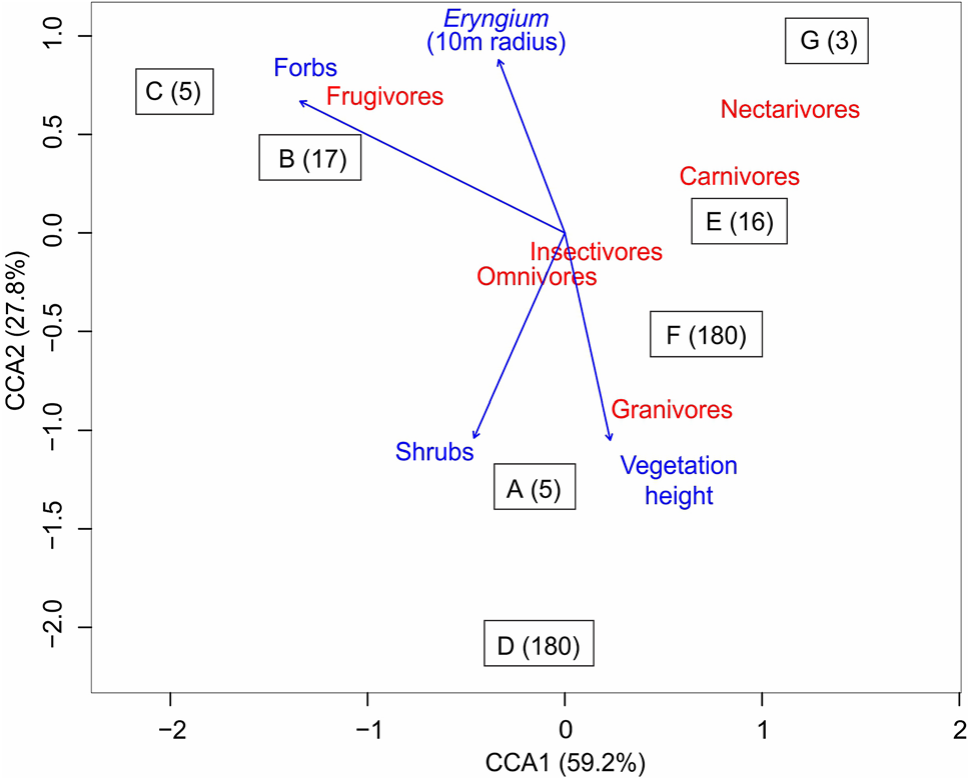
Scatterplot of a Constrained Correspondence Analyses of bird food guilds. The effect of habitat variables (blue arrows; mean vegetation height and abundance of forbs, shrubs, and *Eryngium* in a 10 m radius). In red are the six food guilds used. Respective time since fire (months) of each site: (**A**) (5), (**B**) (17), (**C)** (5), (**D**) (180), (**E**) (16), (**F)** (180), (**G**) (3).

Finally, Constrained Correspondence Analyses also revealed a significant relationship between habitat variables (mean vegetation height and abundance of forbs, shrubs, and *Eryngium* in a 10 m radius) and bird communities, considering both taxonomic (Matrix **W**; ANOVA χ^2^ = 1.048, F = 2.2189, *P* = 0.038; Fig. 5) and functional (Matrix **T**; χ^2^ = 0.5922, F = 13.787, *P* = 0.003, Fig. 6) dimensions. Considering the taxonomic dimention, bird species like *Emberizoides ypiranganus* and *Sporophila melanogaster* were related to taller vegetation and *Eryngium* in sites with longer time since fire. On the other hand, species such as *Cinclodes pabsti* and *Anthus nattereri* were related to the presence of forbs in sites with lower time since fire. Considering the functional dimension, frugivores were related to abundance of forbs and *Eryngium*, whereas granivores were related to taller vegetation in sites with longer time since fire. Nectarivores and carnivores were related to recently-burnt sites and the associated lower vegetation. Finally, insectivores and omnivores showed no clear response pattern to habitat descriptors and seemed to use all types of vegetation.

## Discussion

Here we evaluated the effect of fire disturbance, described by the time elapsed since the last fire event, on habitat and bird community structure descriptors, in Highland Grasslands from South Brazil. We corroborated the general hypothesis that grassland habitat structure and heterogeneity vary according to time since fire. Grasslands with long periods without disturbances usually present an increase in biomass, with homogenization of vegetation structure^39^, which leads to the exclusion of species^14,80^ and to overall diversity losses^14^. Accordingly, we found that sites with longer time since fire presented lower plant species richness for all groups, as well as lower habitat heterogeneity (i.e., high dominance of one or few plant life forms), and overall taller vegetation (which is an indication of increased biomass, although we did not measure this variable directly). These findings corroborate other studies from South American grasslands, providing further evidence that grasslands from this region are a discrete ecological unit that responds similarly to fire disturbance: by reducing the dominance of a few species and life forms that outcompete other taxa in the absence of disturbance^14,15,17^. In grasslands from Argentina, burned sites presented fast recover of plant cover and species richness^15^. In Uruguayan grasslands, fire removed above‐ground biomass of the dominant species (*Saccharum angustifolium*), causing a release of resources, such as space and light, consequently promoting habitat heterogeneity^17^.

Interestingly, plant community responses to time since fire were peaked (or hump-shaped), i.e., plant descriptors showed the highest values in areas with intermediate time since fire (with the exception of the linear response of vegetation height). Peaked diversity-disturbance relationships (DDR) are a common pattern in nature, although not the dominant one: a relatively recent study reviewed almost 200 papers on the subject, and found peaked DDR in ca. 20% of them, including responses of richness, diversity, and evenness^49^. In this study, peaked DDR were more commonly found when the disturbance could be considered ‘natural’ rather than anthropogenic, and intrinsic to the study system. Researchers that find peaked DDR in their study systems often rely on the intermediate disturbance hypothesis (IDH;^50–52^) to explain their patterns, although the mechanisms by which the IDH explain peaked DDR have been severely questioned, both on theoretical and empirical grounds^53^. Nevertheless, we found peaked DDR for most plant community descriptors, providing further empirical evidence that this is a recurrent response pattern of grassland plant communities to disturbance, even though the mechanisms behind this pattern may be different from those postulated in the IDH. Furthermore, the responses of species richness and habitat heterogeneity to fire management relaxation (i.e., lower fire frequency) that we have reported here are similar to what has been reported for grazing relaxation in the same region^41^. This is the expected response of grassland plant diversity to grazing intensity/frequency^54,55^, and it seems that fire disturbance can promote similar response patterns. Differently from other plant descriptors, vegetation height and shrub abundance increased with time since fire (Fig. 3A,D). Sites with longer time since fire are usually dominated by tussock grasses and shrubs (‘shrub encroachment’^43^). Current climatic conditions allow for forest development over grasslands in the study region. In absence of disturbances regimes, shrub encroachment may take place^39,43^, which would ultimately lead to losses of characteristic grassland species^75^. Furthermore, large areas that stay long periods without fire disturbance accumulate woody flammable biomass, and may be subject to a future fire event of greater intensity (often catastrophic), even reaching wetlands and forests^105,106^.

However, the structurally homogeneous and species-poor vegetation that derives from management exclusion seems to provide resource for some specialized birds. Vegetation height has been reported as an important predictor of grassland bird composition^10,107^. Accordingly, we observed some specialized bird species associated with tall vegetation (e.g., *Emberizoides ypiranganus*, *Sporophila melanogaster*, and *Phacellodomus striaticollis*), whereas other species were observed only in shorter vegetation (e.g., *Vanellus chilensis*, *Cinclodes pabsti*, *Anthus* spp.). The differences in species bird composition in distinct vegetation types are due to the biology of each species, which requires different structures of the vegetation to forage, nest, and take refuge. These findings reinforce the importance of habitat heterogeneity, here mediated by fire, for birds with different habitat requirements to colonize^10^.

We partly corroborated our hypotheses relating time since fire to bird community descriptors. Areas with longer time since fire presented lower bird taxonomic diversity and abundance as expected, but no such pattern was found for bird functional diversity. Previous works showed that bird taxonomic diversity and relative abundance decline as disturbance-excluded grasslands become denser with both plant individuals and litter, which ultimately hampers foraging for ground-feeding birds^64^. The overall bird functional composition was predicted by time since fire, but not by habitat structure. The CCA, with fewer selected habitat variables, revealed a significant pattern (Fig. 6). Frugivore birds were associated with forbs and *Eryngium* (Figs. 5,  6)*,* which plays a critical role in the ecology of many bird species (e.g., nesting or foraging^66,85^). We observed the occurrence of Long-tailed Cinclodes (*Cinclodes pabsti*) and Ochre-breasted Pipit (*Anthus nattereri*) in recently burned sites or with an intermediate time since fire. The Ochre-breasted Pipit has been associated with natural grasslands under fire influence^108,109^. In addition, nectarivores and carnivores were also abundant in these sites, probably due to the high abundance of forbs and *Eryngium*, which provide more resources in these shorter grassland sites in comparison with areas with higher and homogeneous vegetation (Fig. 6). Raptors (Accipitridae and Falconidae) are attracted to newly-burned areas for the availability of prey^66^. A study carried in the Highland grasslands observed that the extinction-threatened Saffron-Cowled Blackbird (*Xanthopsar flavus*) is a specialist species restricted to areas under disturbance regimes, with shorter vegetation^110^. Similarly, in Argentina and Uruguay, *X. flavus* selects habitats with short grasses and bare soils that result from livestock grazing^111^. Accordingly, our results indicated that this species was related to recently-burnt areas with shorter vegetation. These findings reinforce that the inclusion of management (i.e., prescribed disturbances) in grassland conservation policies and practices is paramount, since threatened and near-threatened bird species seem to depend on specific habitat configurations that are absent from management-excluded areas.

In an opposite way, granivores were associated with tall vegetation and longer time since fire. This could be related to the higher availability of graminoid seeds for these birds, since sites with longer time since fire had more time to accumulate biomass and produce more seeds. However, we did not sample graminoid species abundance or seed production, therefore this tentative explanation should be considered with caution, and explicitly tested in future research, taking into account the potential negative effects of self-shading processes on seed production^112^. In these sites we found species like the Lesser Grass-Finch (*Emberizoides ypiranganus*) and the Black-bellied Seedeater (*Sporophila melanogaster*), which is a near-threatened species. These species are specialized (and therefore dependent) on tall vegetation structure to nest, forage and seek refuge^71,112–115^. Besides the Black-bellied Seedeater, other granivore species (e.g., Saffron Finch *Sicalis flaveola*, Grassland Yellow-Finch *Sicalis luteola*, and Long-tailed Reed Finch *Donacospiza albifrons*, although the last species also includes insects in its diet) were more abundant in these sites as well, which could also be related to higher seed availability.

Habitat structure was a good predictor of bird community structure, corroborating our last hypothesis. Bird composition, as well as bird taxonomic diversity and abundance, were predicted by habitat variables. Therefore, we corroborate the idea that the response of the bird community to fire disturbance was related to shifts in the relationships of abundance/dominance between plant species in each fire history, an example of community structuring through bottom-up effects^116,117^. Considering the differences in plant descriptors among different times since fire, it seems that the heterogeneity of vegetation structure, and the differential resource availability that follows it, may play an important role in defining patterns of bird communities, as reported before for other ecosystems (e.g.,^9,10,30–32^). The responses of all bird community descriptors to disturbance were linear, which contrasts with the peaked responses found for plant community descriptors. Although areas with longer time since fire showed overall lower bird taxonomic diversity and abundance, they also seemed to benefit granivores birds, a guild that includes important grassland species. Therefore, to maximize conservation results regarding bird species, it is necessary to have grassland patches under different disturbance regimes (i.e., with different fire frequencies), which result in different habitat types for the associated bird species^30,72^. However, seasonal timing and frequency in prescribed fires in grassland management are key issues, especially considering bird species. A fire occurring during the nesting season, a vulnerable time in the life cycle of a bird, may result in loss of active nests or mortality of fledglings^70,72^. Taken together, the responses of plant and bird communities to time since fire we reported here reinforce the well-established close relationship between grassland ecosystems and fire disturbance^1,5,13,14,22,26^.

## Conclusions

Our results indicated that habitat variables related to the grassland vegetation respond to time since fire, and that these habitat variables shape bird community patterns. Plant communities and habitat heterogeneity responded to time since fire in a peaked relationship, whereas bird community descriptors responded linearly. Some bird descriptors responded to time since fire but not to habitat descriptors, suggesting that fire may influence bird communities through direct mechanisms we were not able to detect here. Our results corroborate and expand the bulk of empirical evidence on how South American Grasslands bird communities respond to fire disturbance^71,108,109,112–115^, indicating that a mosaic of areas under different times since fire, which results in a mosaic of diverse grassland habitat structures, seems like the optimal conservation strategy. This conclusion reinforces the idea that the conservation of grassland plant and bird communities must include oriented management, such as prescribed patch burning. However, we emphasize that the conclusions and (especially) management suggestions drawn from our results should be taken with caution, given our relatively small number of spatial replicates. We are confident that the general patterns we presented can be extrapolated to the South Brazilian Highland Grasslands as a unit, but similar studies distributed across the different grassland systems in the region are needed to build more general and widely applicable fire management policies. Nevertheless, periodic prescribed burning has great potential to be used as a tool in the management of natural grassland areas, since it maintains not only the structure and scenic beauty of the grassland landscape, but also the diversity of both plants and birds. We emphasize the importance of habitat heterogeneity, that is, the habitat complexity that results from patches under different fire frequencies, as well as forests when considering the landscape-level mosaic, for the long-term conservation of grasslands in Southern Brazil. Finally, long-term studies are fundamental to determine the periodicity with which fire management should be used for conservation, and the effect of different intensities of grazing combined with fire should be considered in future studies to aggregate financial sustainability to biodiversity conservation.

## Supplementary information

Supplementary Information 1.

Supplementary Information 2.
