# Supplementary material for: The role of fire disturbance on habitat structure and bird communities in South Brazilian Highland Grasslands
Source: Sci Rep. 2020 Nov 12;10:19708. doi: 10.1038/s41598-020-76758-z (PMC7665042; doi:10.1038/s41598-020-76758-z)
Supplement: Supplementary file 1 — Supplementary Information 1. [file 41598_2020_76758_MOESM1_ESM.docx]

# The role of fire disturbance on habitat structure and bird communities in South Brazilian Highland Grasslands

Mariana Beal-Neves^1,2*^, Eduardo Chiarani^2^, Pedro Maria Abreu Ferreira^1^, Carla Suertegaray Fontana^2^

^1^ Laboratório de Ecologia de Interações, Programa de Pós-Graduação em Ecologia e Evolução da Biodiversidade, Pontifícia Universidade Católica do Rio Grande do Sul, building 12, block C, room 111, Ipiranga Avenue 6681 - 90619-900, Porto Alegre, RS, Brasil.

^2^ Laboratório de Ornitologia, Museu de Ciências e Tecnologia, Programa de Pós-Graduação em Ecologia e Evolução da Biodiversidade, Pontifícia Universidade Católica do Rio Grande do Sul, building 40, room 112, Ipiranga Avenue 6681 - 90619-900, Porto Alegre, RS, Brasil.

**E-mail:** mariana.beal@edu.pucrs.br

**Phone:** +55 51 3353 4063

The following xlsx file contains supplementary Tables S1 and S2 that were referred to in the main article.

Table S1. Taxonomic and functional bird communities (matrices W and T). Presents all bird species (n=70) and their respective common name, code and also IUCN threatened species categories. Matrix W with mean species abundance per community with their respective site and time since fire (TSF). Matrix T with classification into food guilds.

Table S2. Habitat variables (matrix E). Presents all habitat variables used in the analyses, their mean values per community and its respective time since fire (TSF).
